# Adult Neurogenesis in Humans- Common and Unique Traits in Mammals

**DOI:** 10.1371/journal.pbio.1002045

**Published:** 2015-01-26

**Authors:** Aurélie Ernst, Jonas Frisén

**Affiliations:** 1 Department of Cell and Molecular Biology, Karolinska Institute, Stockholm, Sweden; 2 Division of Molecular Genetics, German Cancer Research Center (DKFZ), Heidelberg, Germany

## Abstract

New neurons are continuously generated in specific regions in the adult brain. Studies in rodents have demonstrated that adult-born neurons have specific functional features and mediate neural plasticity. Data on the extent and dynamics of adult neurogenesis in adult humans are starting to emerge, and there are clear similarities and differences compared to other mammals. Why do these differences arise? And what do they mean?

## Abstract 2

New neurons generated in the adult brain have been shown in rodents to mediate specific functions, including neural plasticity. This Essay discusses recent work on human adult neurogenesis, examining how it compares to that in other mammals.

## Introduction

For a long time, it was thought that the nervous system is fixed and incapable of regeneration. Although it is indeed true that most neurons in the brain are generated before birth and are never exchanged, it is now well established that new neurons are continuously generated by stem cells in at least two discrete regions in the brain throughout life in most mammals: the hippocampus—a seahorse-shaped structure underneath the cortex that is important for memory formation and cognitive functions; and the olfactory bulb (OB)—a structure located above the nasal cavity that is important for the sense of smell.

At the end of last century, Eriksson, Gage, and colleagues established that new neurons are born in the adult human hippocampus [1]. Only recently, however, has it become possible to acquire quantitative data on the extent and dynamics of adult neurogenesis in humans—by measuring the concentration of the radioactive carbon-14 isotope (^14^C) in genomic DNA. Nuclear bomb tests during the Cold War resulted in an enormous increase in atmospheric ^14^C, which thereafter has declined exponentially, mainly due to uptake by the biotope and diffusion from the atmosphere. The different ^14^C concentrations in the atmosphere at different times is reflected in the human body, and a cell that was born at a certain time will have a ^14^C concentration in its genomic DNA corresponding to the time when the cell was born [2]. Measuring ^14^C in genomic DNA allows retrospective birth dating of cells, and mathematical modeling of such data provides detailed information on the turnover dynamics of a cell population of interest [3]. This research has revealed both that there is more extensive neuronal turnover than many had predicted, and that there is a unique distribution of adult neurogenesis in the adult human brain compared to other mammals. Why is that? And what are the roles of adult neurogenesis in humans?

## Adult Hippocampal Neurogenesis Is Conserved Among Mammals

**Figure 1:**
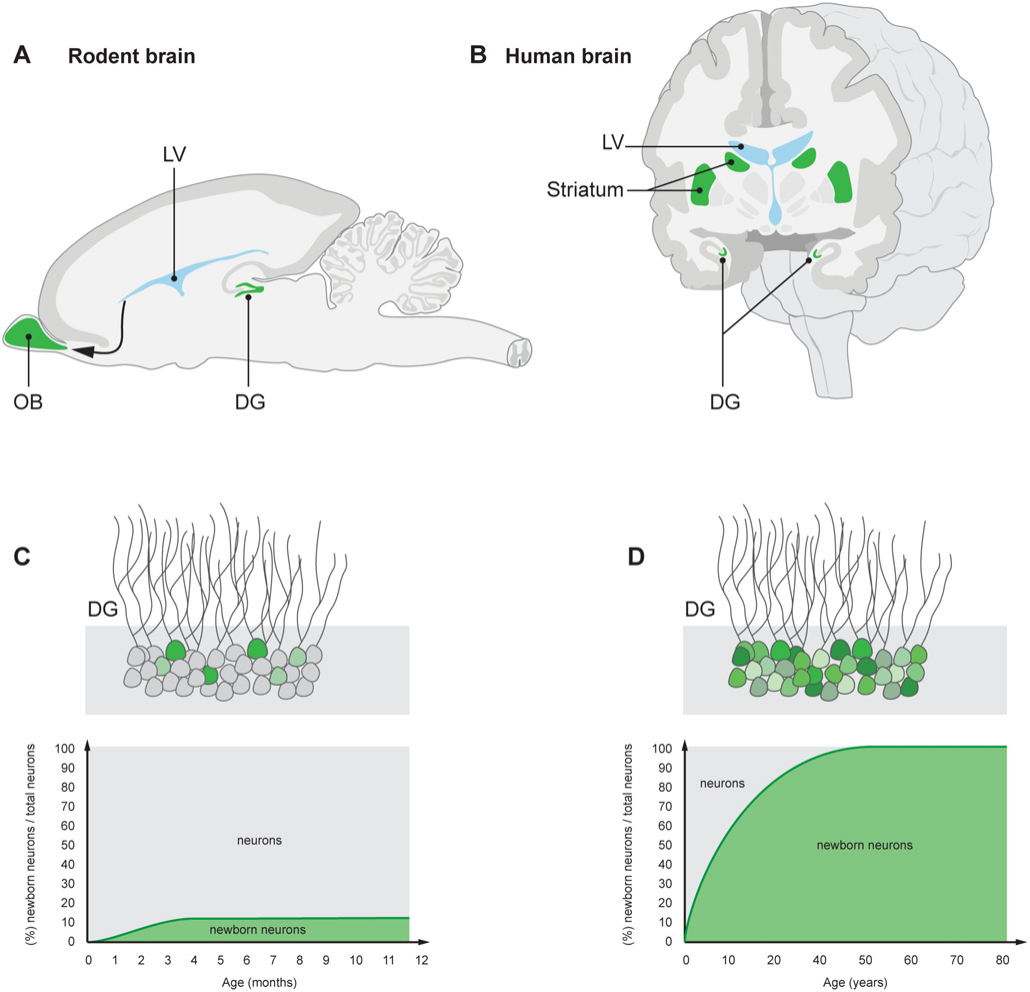
Schematic illustration of adult neurogenesis in the adult rodent and human brain. New neurons are indicated in green. (A) Neuroblasts that are generated in the subventricular zone lining the lateral ventricle (LV) in rodents migrate to the OB, a structure crucial for olfaction, where they integrate as interneurons. (B) Neuroblasts are present in the subventricular zone also in humans, and new neurons integrate in the adjacent striatum, which plays an essential role in movement coordination, procedural learning, and memory, as well as motivational and emotional control. New neurons are continuously generated in the DG of the hippocampus—a brain structure essential for memory and mood control—in both rodents and humans (A, B). A limited subpopulation of DG neurons are subject to exchange in rodents (C), whereas the majority turn over in humans (D) [4–6]. The neurons within the turning over population are continuously exchanged. A value of 100% on the *y*-axis means that all neurons have been replaced since the individual’s birth.

Carbon dating demonstrated that hippocampal neurons are generated at comparable rates in middle-aged humans and mice [4]. However, humans present a somewhat different pattern of adult hippocampal neurogenesis as compared to rodents (Fig. 1). The vast majority of the neurons in the dentate gyrus (DG), the subdivision of the hippocampus with neuronal turnover, is subject to exchange in humans, compared to approximately 10% in mice [4–6]. Moreover, humans show a less pronounced age-dependent decline in hippocampal neurogenesis during adulthood compared to mice [4]. Adult-born hippocampal neurons are more likely to be lost than the neurons born during development in humans [4]. Whether this is also the case in other mammals has not been directly investigated, but data from mice is consistent with this notion [7].

Image credit: Mattias Karlén.

## Adult Neurogenesis in the Subventricular Zone and Olfactory Bulb

Neuronal precursor cells, or neuroblasts, are produced not only in the hippocampus but also in the subventricular zone of the LV wall in adult humans, like in other mammals. The density of neuroblasts and the dynamics of its decline with age are very similar between the hippocampus and subventricular zone in humans [8–10]. However, whereas most features of adult hippocampal neurogenesis appear rather highly evolutionarily conserved, there are large differences between humans and other mammals in the output of new neurons from the subventricular zone. In rodents and nonhuman primates, these neuroblasts migrate to the OB (Fig. 1) [11, 12].

Humans appear unique among mammals in that there is negligible, if any, addition of new neurons in the OB after the perinatal period. This conclusion is based on the very few neuroblasts that can be found in the adult human rostral migratory stream, the migratory path from the subventricular zone to the OB, and carbon dating of OB neurons [9, 13]. Although it is not possible to conclude a complete absence of adult OB neurogenesis in adult humans, carbon dating sets the limit to what could go undetected to less than 1% of the OB neurons being exchanged over 100 years [13]. One study reported very large numbers of proliferating cells in the human subventricular zone and rostral migratory stream, implicating substantial adult human OB neurogenesis [14], but only very small numbers of neuroblasts and no evidence of new mature neurons were found in subsequent studies [9, 13, 15].

## Adult Striatal Neurogenesis Is Most Pronounced in Humans

The striatum is a forebrain structure underneath the cortex and is involved in regulating motor behaviors and responses to rewarding and aversive stimuli. In rodents, the vast majority of neurons generated in the subventricular zone integrate in the OB. There are, however, studies suggesting the postnatal generation of small numbers of striatal interneurons in mice, rats, and rabbits [16–18]. Adult neurogenesis was also reported in the striatum of untreated and sham-operated adult nonhuman primates [19–21]. In squirrel monkeys, a subset of newborn cells was found to deviate from the rostral migratory stream. Instead of reaching the OB, these cells were shown to migrate into the part of the ventral striatum called the olfactory tubercle, where they displayed a mature neuronal phenotype [20].

In humans, neuroblasts are not restricted to the LV wall, but are also present adjacent to this neurogenic niche, in the striatum (Fig. 1) [22]. Detection of the thymidine analog iododeoxyuridine in striatal interneurons, which allows prospective labeling of dividing cells and identification of their progeny, showed the generation of this cell type in adults. Retrospective birth dating of striatal neurons confirmed the postnatal generation of interneurons [22].

It appears likely that the neuroblasts and new neurons in the adult human striatum derive from the neighboring subventricular zone. One major difference in adult neurogenesis between rodents and humans may thus be the direction of neuroblast migration from the subventricular zone, with the OB being the principal destination in most mammals. It is also possible that new striatal neurons derive from local cells within the parenchyma [23], perhaps in addition to those from the subventricular zone.

That substantial adult striatal neurogenesis is seen only in humans, and possibly some nonhuman primates, makes it challenging to study this process in commonly used experimental animals. However, blocking the Notch signaling pathway in astrocytes in the intact striatum of mice triggers neurogenesis in the otherwise intact striatum [23], potentially offering a suitable model to assess the functional role of adult striatal neurogenesis in an experimentally more tractable organism.

## Neurogenesis in the Adult Neocortex?

The potential addition of neurons to the adult mammalian neocortex has been a source of controversy. In rodents and nonhuman primates, some reports have suggested that neurogenesis continues in the adult neocortex [16, 24, 25]. Other studies have not detected neurogenesis in this region under physiological conditions [26–28], or have argued for a transient existence of adult-born cortical neurons [29]. In humans, we showed that neocortical neurogenesis is restricted to development [30] and found that cortical neurons are as old as the individual even after stroke [31]. Klempin and colleagues demonstrated that cells expressing the commonly used neuroblast marker doublecortin (DCX) in the mouse piriform cortex (part of the olfactory cortex) were strictly postmitotic [32], and expression of neuroblast markers alone can only be regarded as an indication of potentially ongoing neurogenesis.

## Evolutionary Perspectives on Adult Neurogenesis

**Figure 2:**
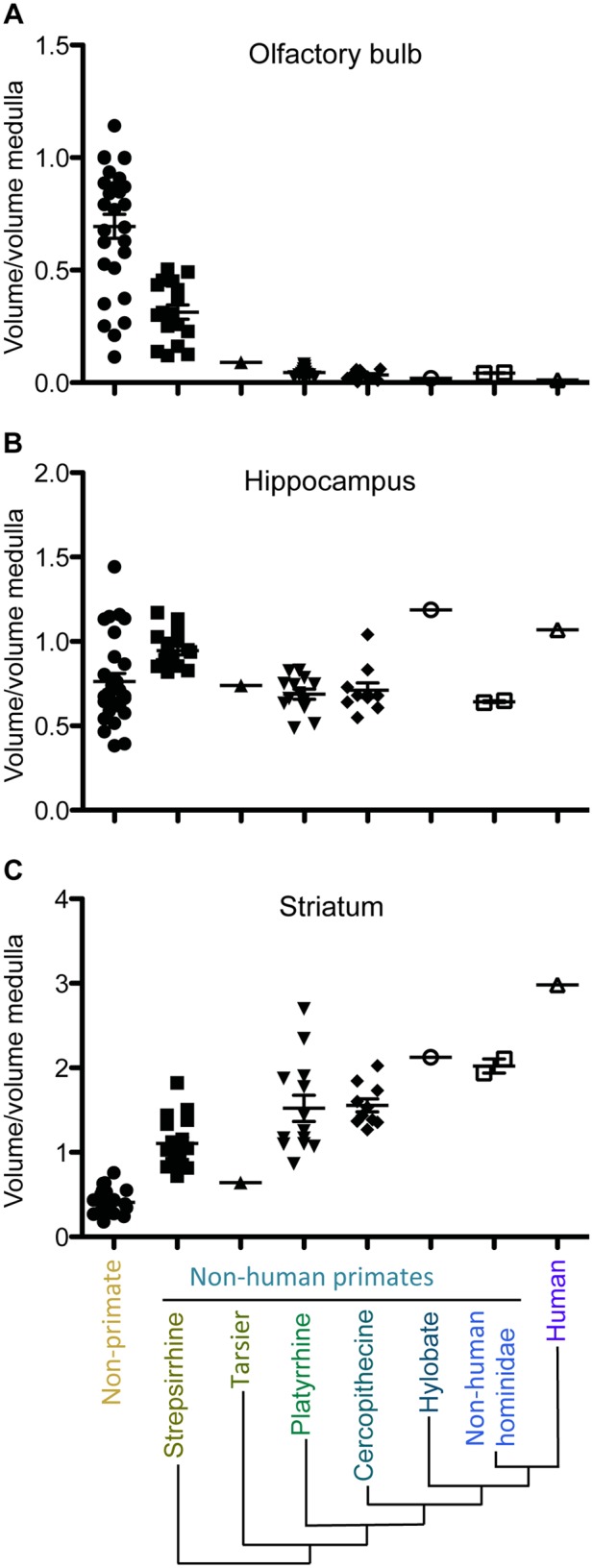
Proportional OB (A), hippocampal (B), and striatal volumes (C). Species are grouped according to major phylogenetic classes varying in phylogenetic distance from humans: nonprimates first (e.g., shrews, tenrecs, hedgehogs), then *Strepsirrhine* (e.g., lemur), *Tarsier*, *Platyirrhine* (e.g., New World monkey), *Cercopithecine* (e.g., baboon, macaque), *Hylobate* (e.g., gibbon), Nonhuman hominidae (e.g., chimpanzee, gorilla). The proportional volume of the OBs decreases across primate species. Humans display the most pronounced reduction in OB volume. Hippocampal volumes appear to maintain their proportions across species, whereas proportional striatal volumes increase with evolution. The proportions of regional brain volumes are calculated as proportions of medulla volumes, because no grade shifts in the relationship between medulla volume and body size are observed [34]. Volumetric measurements are from Stephan et al. [48].

What could explain the divergent patterns of adult neurogenesis in distinct regions of the mammalian brain? New neurons, as well as changes in the proportion and organization of particular brain structures, offer a selective advantage to individuals by giving them the cognitive adaptability necessary to conquer diverse ecological niches [33]. In general, increasing the size of a brain region enhances the associated functional domains [34]. A decrease in olfactory abilities with evolution is well documented and linked to a reduced dependence on olfaction. This functional regression is associated with a decrease in OB volume across phylogenetic groups, and most extremely in humans (Fig. 2A) [34]. In contrast, relative hippocampal volumes remain rather constant across species, which supports the notion that hippocampal memory seems to be necessary for the success of an organism, regardless of its environmental niche (Fig. 2B) [34]. The neostriatum, comprising the caudate nucleus and the putamen, is a phylogenetically new component of the brain. Over the course of evolution, the striatum enlarged in parallel with the cerebral cortex; it is particularly well developed in higher mammals, including humans (Fig. 2C). This proportional increase of the striatum with evolution implies a heavier reliance on movement coordination, cognition, and emotions.

**Figure 3:**
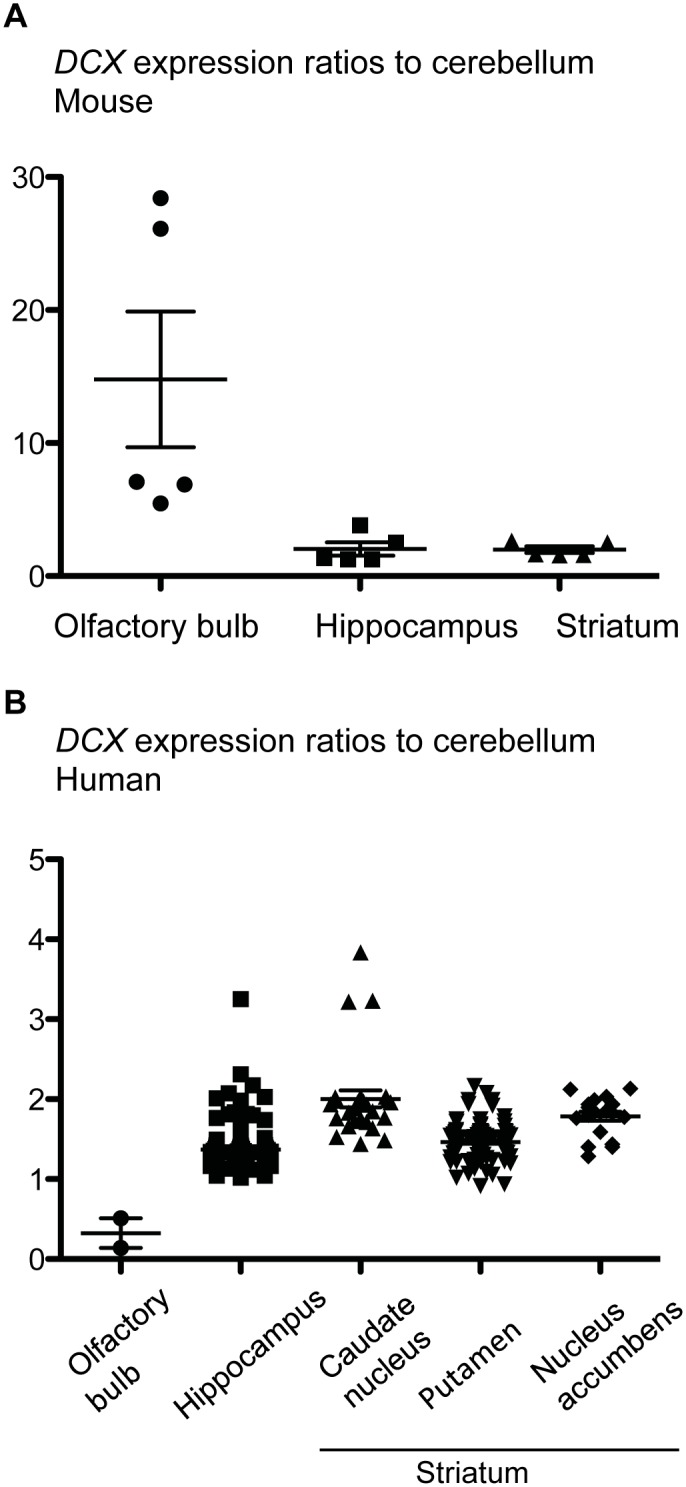
Expression levels of the neuroblast marker *DCX* in the OB, hippocampus, and striatum of adult mice (A) and humans (B) normalized to the expression levels in the non-neurogenic adult cerebellum. In mice, *DCX* expression is much higher in the OB than in the hippocampus and striatum. In humans, only background levels are detected in the OB, whereas higher *DCX* expression levels are reached in the human hippocampus and striatum. mRNA expression was measured by in situ hybridization, expression profiling, and RNA sequencing. Data are from geo (GSE 2361, GSE 45878, GSE 46706, GSE 1133, GDS1490, GDS182) and from the Allen Brain Atlas. The data points for the human OB show pooled values for several donors.

**Figure 4:**
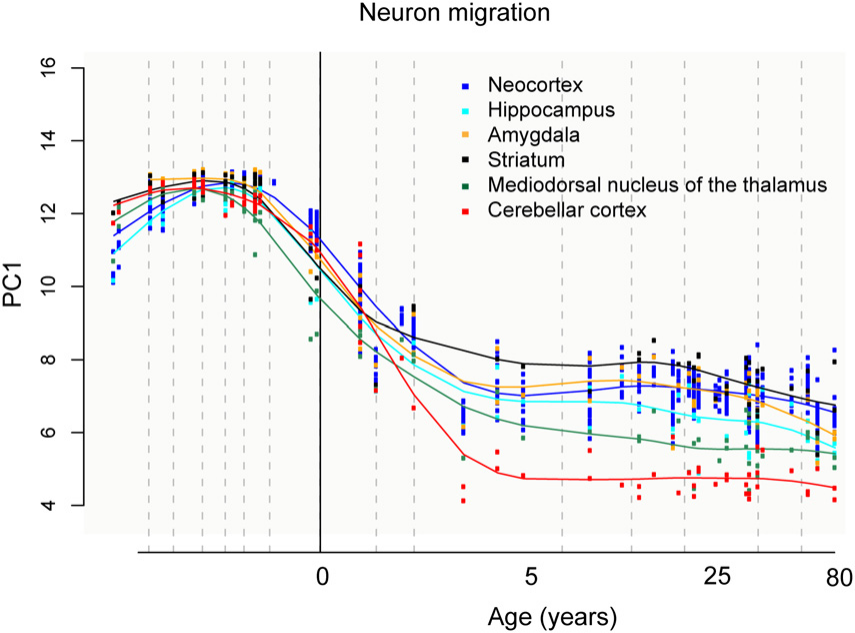
Transcriptome-based expression trajectories of genes associated with neuronal migration in the human striatum compared to other brain regions. *Y*-axis, first principal component (PC) value for gene expression. Expression levels of 100 genes reported to be associated with neuronal migration are taken into account (see Kang et al. for details on the statistical methods for the principal component analysis and exhaustive list of genes included). *X*-axis, subject age in years. Data from Kang et al. [35].

Indications of the extent of adult neurogenesis in a specific brain region can be inferred from the expression level of markers for immature neurons. In mice, mRNA expression of *DCX* is much higher in the OB than in the hippocampus and striatum (Fig. 3A). In contrast, in humans, only background levels are detected in the OB. *DCX* expression levels are comparable in the human hippocampus and in the putamen; they reach the highest values in the caudate nucleus and in the nucleus accumbens (which is part of the ventral striatum) (Fig. 3B). When taking into account additional markers of immature neurons, genes associated with neuronal migration show the highest expression in the striatum in adult humans, as compared to other brain regions (Fig. 4).

These observations are in line with the evolutionary changes in volume and functional performance of the OB, hippocampus, and striatum described above. In the human hippocampus, *DCX* transcript levels correlate closely with the number of neuroblasts [35], which in turn shows a strong association with the number of newly generated neurons [4]. Estimates of the extent of neurogenesis based on *DCX* expression support the lack of detectable adult OB neurogenesis in humans [13] and the comparable neuronal turnover rates in the adult human hippocampus and striatum [4, 22].

## Potential Functions for Adult Neurogenesis in Humans

There is continuous generation of hippocampal neurons throughout life in humans, to an extent comparable to adult neurogenesis in the mouse. Therefore, the level of neurogenesis in the adult human hippocampus may be sufficient to contribute to brain function, and might have similar functions in cognitive adaptability as in rodents [4].

The functional significance of adult striatal neurogenesis remains to be established. Even though the longevity of the adult-born neurons argues for a probable functional integration, it is still to be determined whether the extent of postnatal neurogenesis may be sufficient to be utilized for therapeutic purposes (Box 1). However, low rates of neurogenesis under homeostatic conditions can be increased in response to pathological conditions, and the continuous addition of small numbers of new neurons to the injured striatum over long periods can add up to a significant amount of cells [36]. Furthermore, even a limited number of new neurons can potentially have a substantial functional impact, provided they integrate at critical points in the existing circuitry. Newly generated neurons possess unique properties (e.g., enhanced synaptic plasticity) that allow them to perform special tasks for a limited time after their birth [37, 38].

**Box 1. Adult Neurogenesis and Striatal Disorders**

The identification of a subset of neurons that is renewed in the adult human striatum raises the question whether this process can be taken advantage of for therapeutic purposes. A wide variety of disorders may affect the striatum, among which are acquired conditions such as stroke, but also genetically inherited disorders such as Huntington’s disease. Increasing the generation or promoting the survival of new neurons might offer an attractive possibility in some cases.

In response to stroke, striatal neurons are generated from the subventricular zone in rodents and nonhuman primates [21, 49–52]. At least in mice, new neurons are also generated by local astrocytes within the striatum after stroke [23]. Two groups showed an increase in proliferation and neuroblast production after stroke in the human subventricular zone [53, 54], which may indicate increased adult neurogenesis in this situation. However, it is still unclear whether neuroblasts generated after stroke can survive and give rise to mature neurons in the human striatum. Retrospective birth-dating will allow quantification of the extent of neurogenesis after striatal stroke and to discern whether this process can be utilized to provide novel treatment options.

Huntington’s disease is a neurodegenerative disorder that primarily affects striatal neurons. Increased cell proliferation and neuroblast production have been reported in the subventricular zone of Huntington’s disease patients [55, 56]. However, postnatally generated striatal neurons are depleted in advanced stages of the disease [22]. It is conceivable that Huntington’s disease triggers an increase in proliferation to compensate the loss of striatal neurons, but that the neuroblasts die before producing differentiated neurons, or give rise to mature neurons that undergo apoptosis shortly after their generation.

Along with stroke and Huntington’s disease, a number of other disorders and conditions also interfere with striatal neuron function, including Parkinson’s disease, schizophrenia, and addiction. Certain subtypes of neurons in the striatum appear to be more resistant to disease [57, 58]. Investigating how striatal neurogenesis is affected in pathological situations and which factors promote the renewal of striatal neurons may facilitate the development of therapeutic approaches.

Which human- or primate-specific striatal functions could necessitate postnatal neurogenesis? The human striatum is now recognized to play a key role for higher cognitive functions, in particular “cognitive flexibility”, the ability to adapt behavioral goals in response to changing contextual demands [39, 40]. Striatal amphetamine-induced dopamine release predicts individual differences in cognitive flexibility [41]. In children, striatal volume was shown to be associated with neurocognitive performance [42, 43]. Primates possess a number of unique cognitive specializations, some of them being supported by the striatum. In nonhuman primates, the relative striatal volume correlates with the rate of social play behavior across species, suggesting a coevolution of traits [44].

The striatum also has a decisive function in the planning and modulation of movement, which poses the question whether postnatal neurogenesis in the striatum might be required for certain human- or primate-specific motor tasks. In Huntington’s disease, striatal atrophy—which parallels neuronal loss—begins many years before movement abnormalities appear, and the decrease of the striatal volume predicts when motor onset will occur [45].

In addition to the coordination of cognitive and motor functions, the striatum is involved in reward, motivation, and pleasure. In animals, the mesolimbic reward system reinforces biologically vital behaviors, such as eating, sex, or caring for offspring. Over the course of evolution, additional factors became important for successful survival. Humans have the ability to experience pleasure and reinforcing behaviors from more abstract stimuli, such as art or money, which also implicate the mesolimbic striatal area. People differ widely in their willingness to postpone immediate gratification to pursue long-term goals, i.e., how much they discount delayed rewards. Neural activity in the ventral striatum when subjects are asked to think about the future predicts delay discounting [46]. The mesolimbic striatal system also mediates emotion associated with art; specifically, reward value for music can be coded by activity levels in the nucleus accumbens, whose functional connectivity with auditory and frontal areas increases as a function of increasing musical reward [47].

Currently, we can only speculate about the potential functions of adult striatal neurogenesis. Striatal adult neurogenesis may have evolved to provide specific types of neural plasticity in humans and possibly in nonhuman primates. Strategies to modulate postnatal neurogenesis in the striatum of nonhuman primates and evaluate the cognitive, motor, and emotional response might help to uncover what new neurons do in old brains.

## Abbreviations

^14^C: carbon-14 isotope
DCX: doublecortin
DG: dentate gyrus
LV: lateral ventricle
OB: olfactory bulb
PC: principal component
